# High Myopia and Thickness of Extraocular and Masticatory Muscles—7T MRI, Preliminary Study

**DOI:** 10.3390/jcm12124166

**Published:** 2023-06-20

**Authors:** Grzegorz Zieliński, Anna Matysik-Woźniak, Anna Pankowska, Radosław Pietura, Robert Rejdak, Kamil Jonak

**Affiliations:** 1 Department of Sports Medicine, Medical University of Lublin, 20-093 Lublin, Poland; 2 Chair and Department of General and Pediatric Ophthalmology, Medical University of Lublin, 20-093 Lublin, Poland; anna.wozniak@umlub.pl (A.M.-W.); robert.rejdak@umlub.pl (R.R.); 3 Department of Radiography, Medical University of Lublin, 20-093 Lublin, Polandradoslaw.pietura@umlub.pl (R.P.); 4 Department of Clinical Neuropsychiatry, Medical University of Lublin, 20-093 Lublin, Poland

**Keywords:** high myopia, myopia, masticatory muscles, TMJ, extraocular muscles, MRI, 7T, temporalis muscle, optometry

## Abstract

(1) Background: Myopia is one of the most common refractive errors in the world. The aim of this study was to evaluate the transverse dimensions of selected masticatory muscles (temporalis muscle and masseter muscle) versus the transverse dimensions of selected extraocular muscles (superior rectus, inferior rectus, medial rectus and lateral rectus) in emmetropic and high myopic subjects. (2) Methods: Twenty-seven individuals were included in the analysis, resulting in 24 eyeballs of patients with high myopia and 30 eyeballs of emmetropic subjects. A 7 Tesla resonance was used to analyze the described muscles. (3) Results: Statistical analysis showed differences in all analyzed extraocular muscles and all analyzed masticatory muscles between emmetropic subjects and high myopic subjects. In the high myopic subject group, statistical analysis showed four correlations. The three negative correlations were between the lateral rectus muscle and an axial length eyeball, refractive error and an axial length eyeball, and the inferior rectus muscle and visual acuity. The positive correlation was between the lateral rectus muscle and the medial rectus muscle. (4) Conclusions: The high myopic subjects are characterized by a larger cross-sectional area of extraocular muscles and masticatory muscles compared to the emmetropic subjects. Correlations were observed between the thickness of the extraocular muscles and the masticatory muscles. The lateral rectus muscle was related to the length of the eyeball. The phenomenon requires further study.

## 1. Introduction

Myopia is one of the most common refractive errors in the world [1], consisting of the optical system of the eye incorrectly focusing light rays [2,3]. Myopia develops primarily in childhood [4]. The etiology of myopia is multifactorial and incompletely understood. The formation of myopia may be influenced by genetic factors [5,6] and environmental factors (urbanization, level of education, time spent outdoors, physical activity, etc.) [7,8,9]. It is suggested that interactions between genetic and environmental factors may have a significant impact on high myopia [7].

High myopia is defined as a refractive error ≤−6.00 Diopters (D) [3] or axial length greater than 26 mm [10]. According to estimates, 2.7% of the world’s population was affected by high myopia in 2000. Research predicts that this number will rise to 9.8% of the world’s population in 2050 [1]. High myopia is associated with many consequences: macular degeneration, maculopathy, risk of retinal detachment, glaucoma and cataracts [11].

**Figure 1:**
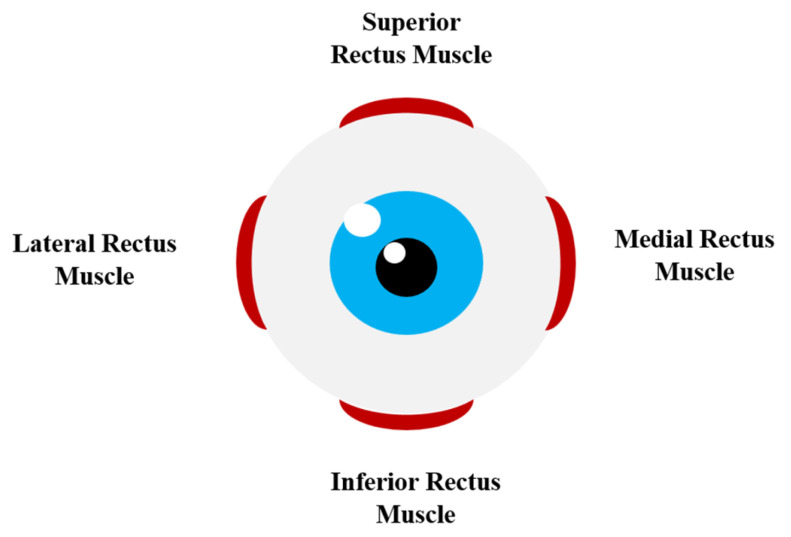
Presentation of the tested extraocular muscles.

The ability to see in different directions is due to the different course and attachment site of the extraocular muscles. Six extraocular muscles are responsible for eye movement:

- Superior rectus (primary action: elevation; secondary action: incyclotorsion; tertiary action: adduction);
- Inferior rectus (primary action: depression; secondary action: excyclotorsion; tertiary action: adduction);
- Medial rectus (primary action: adduction);
- Lateral rectus (primary action: abduction) (Figure 1);

- Superior oblique (primary action: incyclotorsion; secondary action: depression; tertiary action: abduction);
- Inferior oblique (primary action: excyclotorsion; secondary action: elevation; tertiary action: abduction) [12].

These muscles work in synergy and are subject to constant contraction and diastole according to Sherington’s law (any increase in nerve excitation of the agonist muscle must be accompanied by a concomitant decrease in the activity of the antagonist muscle) and Hering’s law (if one muscle performs a movement, the identical muscle in the other eye performs an identical movement) [13].

Researchers suggest connections between the musculo-fascial system and the organ of vision [14,15]. They suggest hypothetical connections along the fascial pathway. The eyeball moves within the Tenon’s capsule, which extends from the optic nerve up to the corneal limbus, and the Tenon’s capsule itself fuses with the extraocular muscles [16]. Subsequently, via the levator palpebrae superioris muscle and the orbicularis oculi muscle of the eye, it connects to the superficial musculoaponeurotic system (SMAS) [17]. Then, using the fascial pathway, it connects to other musculo-fascial structures of the body [18]. According to a recent study (2022), correlations have been observed between muscle bioelectrical activity and the length of the eyeball on the same side [19]. In addition, another study (2022) observed that bioelectrical activity within the anterior temporalis muscle appears to be related to eye length, retinal thickness and choroidal thickness [18].

In view of these observations, the present study was designed. To the best of our knowledge, this was the first study of its kind for which the main aim was to evaluate the transverse dimensions of selected masticatory muscles (temporalis and masseter muscles) versus the transverse dimensions of selected extraocular muscles (superior rectus, inferior rectus, medial rectus and lateral rectus) in emmetropic and high myopic subjects.

## 2. Materials and Methods

The study was conducted with the approval of the local Bioethics Committee (Bioethics Committee of the Medical University of Lublin, Poland, approval number KE-0254/183/2021). Subjects enrolled in the study knew the purpose of the study and knew that they could opt out at any time during the study. Each participant gave informed consent to participate in the study.

Inclusion criteria for the study: age over 18; no refractive error; a visual acuity of 1.0 for the control group and a refractive error greater than −6 D for the study group [3]; an axial length of the eyeball for the control group in the range of 23–24 mm [20] and for the study group greater than 26 mm [10].

The following exclusion criteria were used: farsightedness, myopia of less than −6.00 D [3], optic nerve disease, anterior eye disease, metal implants in the head and neck region, pregnancy, cancer (regardless of type and location), surgical procedures in the head and neck region within the last 6 months, head or neck pain on the day of the study, intraocular pressure of more than 20 mmHg, strabismus, cataract, and retinal detachment.

Individuals in the study group were patients of the Chair and Department of General and Pediatric Ophthalmology who presented to the unit for high myopia. Emmetropic subjects were those who volunteered for the study among local citizens. Both groups included residents of Lublin (a city in eastern Poland with a population of more than 300,000). The patients were Caucasian and had received primary and higher education.

After applying exclusion criteria to the 29 subjects enrolled, 27 subjects were included in the study. The group was divided into a study group (high myopic subjects, *n* = 12, men = 3, women = 9) and a control group (emmetropic subjects, *n* = 15, men = 9, women = 6). A total of 30 eyeballs were analyzed in the group of emmetropic subjects, and 24 eyeballs were analyzed in the group of high myopic subjects. A chi-squared test was used for 2 × 2 contingency tables alone, and Fisher’s exact test was used to analyze group sizes and the number of men and women in the groups [21,22]. The obtained result in chi-squared test for 2 × 2 contingency tables was not statistically significant (chi-square 2.74, df = 1, *p* = 0.098), and the obtained result in Fisher’s exact test was not statistically significant (*p* = 0.104) (Table 1).

All participants underwent a comprehensive ophthalmologic examination. Visual acuity was examined on a Snellen chart, refractive error was determined using a Topcon KR-800 autokeratorefractometer test (Topcon Co. Tokyo, Japan), and eyeball length was determined using an IOL Master 500 (Carl Zeiss Meditec, Jena, Germany). The 1% tropicamide [23] was instilled before the examination of refractive errors in order to control accommodation and to assess the patients’ retina. The structures of the anterior segment of the eyeball were evaluated in a slit lamp, and, at the end of the study, the intraocular pressure was checked with a Tono-Pen XL (Medtronic Solan, Jacksonville, FL, USA). All subjects had an intraocular pressure of less than 20 mmHg [18].

The 7T MRI machine at Ecotech Complex Lublin captured a three-dimensional inversion recovery-prepared spoiled gradient echo (3D-SPGR “BRAVO”) image using a 32-channel coil. The imaging parameters were as follows: the field of view was 220 × 220 × 180 mm, the acquisition matrix was 256 × 256 × 180, and the images were reconstructed to a final matrix size of 512 × 512. Consequently, the resulting voxel size was 0.43 × 0.43 × 1 mm. The specific imaging parameters used were TE (echo time) of 2.6 ms, TR (repetition time) of 6.6 ms, TI (inversion time) of 450 ms, and a flip angle of 12 degrees. The bandwidth was +/−31.25 kHz, and parallel imaging (ARC) with a factor of 2 was employed.

Due to the high field inhomogeneity in 7T MRI, each structural volume was corrected for intensity error using algorithms of the unified segmentation process in MATLAB software—SPM 12 ([24]; ver. MATLAB R2018A, Mathworks, Inc, Natick, MA, USA). The brain segmentation procedure was performed using the “recon-all” function in FreeSurfer software [25]. To ensure stable processing, the voxel size was set to 0.5 mm^3^ from the native size. The number of surface layers was set to 100 and implemented into recon-all as a flag function in cm. In the next plug, a procedure was performed to process the entire image, including normalization of signal intensity, removal of artifacts to extract cranial regions in normalized space and automatic segmentation. After preprocessing, the quality of the images was assessed by a radiologist. Each slice was checked for artifact removal errors, segmentation, normalization, bone surface changes and topological defects according to FreeSurfer guidelines. The appropriate preprocessing steps were then repeated for participants whose images required editing. Morphometric assessments of facial muscles were performed using the T1 protocol.

Muscles were selected for thickness assessment. The extraocular muscles (superior rectus, inferior rectus, medial rectus and lateral rectus) were chosen because they are the most important four muscles responsible for eye movements [12]. In addition, a recent study (2021) showed that parameters of rectus muscles are connected with myopia [26]. The muscles of the masticatory organ (temporalis and masseter muscles) were chosen because they are the largest muscles affecting the temporomandibular joint [15]. In addition, the temporalis muscle is often associated with tension headaches [27,28]. Some studies have linked more frequent headaches with myopia [29,30].

Statistical analysis was performed using IBM SPSS Statistics 13.3. In the first stage, the results were analyzed for normality of distribution using the Shapiro–Wilk test and the Kolmogorov–Smirnov test (with Lillierfors correction). Due to the small sample size and age of subjects with a distribution different from normal, non-parametric tests were used for further analysis. Mann–Whitney U (Z) tests were used for analysis between groups, and Spearman’s rho test was used for correlation analysis. As recommended by Sullivan and Feinn [31], effect size was determined according to Cohen’s classification as small (d = 0.2), medium (d = 0.5) and large (d ≥ 0.8) [32]. Additionally, statistical significance was set at *p* ≤ 0.05.

## 3. Results

Statistical analysis showed that the groups (emmetropic subject and high myopic subject) differed in age, visual acuity and eyeball length (Table 1).

**Figure 2:**
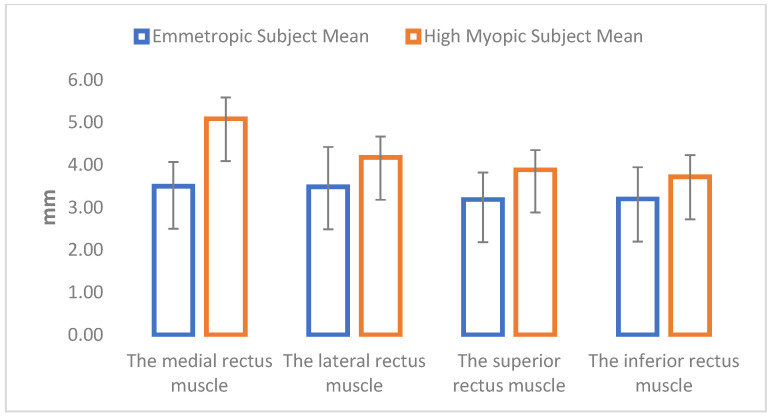
Graphic representation of the differences in the thicknesses of the extraocular muscles between the groups.

**Figure 3:**
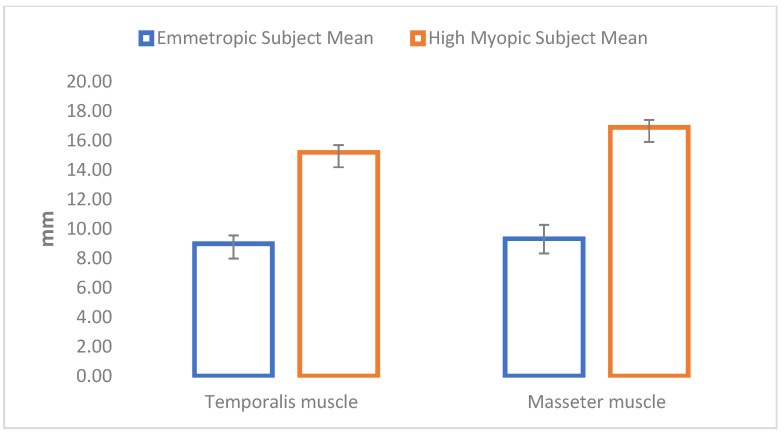
Graphic representation of the differences in the thicknesses of the masticatory muscles between the groups.

Statistical analysis showed differences in all analyzed extraocular muscles and all analyzed masticatory muscles between emmetropic subjects and high myopic subjects. High myopic subjects showed increased muscle thickness in all parameters (Table 2; Figure 2 and Figure 3).

The statistical analysis of the emmetropic subjects showed five correlations. Positive correlations were shown between the lateral rectus muscle and the medial rectus muscle, the inferior rectus muscle and the superior rectus muscle, the temporalis muscle and the superior rectus muscle, the temporalis muscle and the inferior rectus muscle, and the masseter muscle and the medial rectus muscle (Table 3).

In the high myopic subject group, the statistical analysis showed five correlations. There were three negative correlations between the lateral rectus muscle and an axial length eyeball, refractive error and an axial length eyeball, and the inferior rectus muscle and visual acuity. One positive correlation was between the lateral rectus muscle and the medial rectus muscle, and another positive correlation was between the masseter muscle and the temporalis muscle (Table 4).

## 4. Discussion

To the best of our knowledge, this was the first study of its kind for which the primary objective was to evaluate the transverse dimensions of selected masticatory muscles (temporalis muscle and masseter muscle) versus the transverse dimensions of selected extraocular muscles (superior rectus, inferior rectus, medial rectus and lateral rectus) in emmetropic and high myopic subjects.

The specified thickness of the extraocular muscles was similar to studies conducted on Turkish [33], Thai [34] and Indian [35] populations, while there were differences between our reported thicknesses and those reported in studies conducted on Italian [36] and Chinese [37] populations. When considering the thickness of the muscles in people with high myopia, similar values to the studies of Pierro et al. of the medial rectus and lateral rectus muscles [36] were noted, and differences were observed between the superior rectus and inferior rectus muscle. Differences between emmetropic and myopic subjects concerning muscle changes have also been reported in studies [26,36].

Analyzing the masticatory muscles, the resting thickness of the temporalis muscle in those without a refractive error was 12.25–13.18 mm, and the resting thickness in those with a refractive error was 12.20 mm (the results were not statistically significant). In one study, the thickness of the masseter muscle at rest in subjects without a refractive error was 11.94–12.43 mm, and in subjects with a refractive error, it was 12.42–12.69 mm (the results were not statistically significant) [18]. To date, this has been the only study to compare muscle thicknesses between people with a refractive error (low myopia) and those without a refractive error [18]. In the results of the authors’ study, it was noted that the thickness of the temporalis and masseter muscles differed significantly between emmetropic and high myopia subjects (Table 2). Hypothetically, the differences in results between our result and the results from this mentioned research [18] could be due to the magnitude of the refractive error. In our study, there were people with high myopia (above −6.00D).

In subjects with high myopia, the authors’ study found four significant relationships. In our opinion, the most significant correlations are between the lateral rectus muscle and an axial length eyeball and the inferior rectus muscle and visual acuity (Table 4). A study by Vasudev et al. noted that medial rectus muscle parameters showed a significant relationship with myopia [26]. The cited authors point out that the role in myopiaogenesis of this muscle needs further research. In our study, a correlation with the lateral rectus muscle was observed. This phenomenon shows a possible direct link between extraocular muscles and the development of myopia. Another connection that needs to be noted is the observed correlation between visual acuity and the inferior rectus muscle. This connection may be related to the hypothesized influence of the fascial network on the eyeball [15,18]. From the optic nerve, a thin connective tissue membrane (Tenon’s capsule) surrounds the extraocular muscles, fuses with the sclera and passes into the subconjunctival connective tissue [16,18].

It is noteworthy that correlations between the masticatory and studied extraocular muscles occurred only in the emmetropic subject group (Table 3). The most likely connection here is via the fascial isthmus. The Tenon’s capsule, already described, connects to the levator palpebrae superioris, then to the orbicularis oculi muscle and, subsequently, with the superficial musculoaponeurotic system (SMAS) [17]. Studies to date have hypothesized linking changes in the bioelectrical activity of the masticatory muscles to structural changes in the extraocular muscles [15,18,19]. These connections underscore the importance of collaboration between specialists from different disciplines.

Future research should focus on further analyzing the possible connections between the extraocular muscles and other muscles of the human musculoskeletal system. In addition, future research should analyze the possible influence of the aforementioned muscles on the development of the refraction error and its progression. Specialists working with patients with refractive error and patients with craniofacial muscle problems should take into account the possible interaction of the two systems.

The present study has the following limitations. We studied Caucasian subjects. Because of the severity of myopia in different regions of the world, it is worth repeating the study with people of other races [38,39]. We studied adults; it is worth repeating the study on children. In our study, we tested patients with high myopia. For this reason, it is worth repeating the study on people with low myopia. The last limitations are that we studied groups that differed in age and that the study was conducted on a small number of subjects.

## 5. Conclusions

The high myopic subjects are characterized by a larger cross-sectional area of extraocular muscles and masticatory muscles compared to the emmetropic subjects. Correlations were observed between the thickness of the extraocular muscles and the masticatory. The lateral rectus muscle was related to the length of the eyeball. This phenomenon requires further study.

## Figures and Tables

**Table 1:** Intergroup comparison.

	Emmetropic Subjects		High Myopic Subjects				
	Mean	SD	Mean	SD	Test		*p*
*n* Eyeballs	30		24				
Age	33.71	6.49	39.92	11.84	Z	−2.08	0.04
Visual Acuity Right	1.0		0.6	0.4	Z	−2.30	0.02 *
Visual Acuity Left	1.0		0.4	0.3	Z	−3.31	0.00 *
Refractive Error Right			−9.75	5.25			
Refractive Error Left			−11.50	6.25			
Axial Length Eyeball	23.58	0.36	27.14	2.31	Z	−6.12	0.00 *

* Significant difference.

**Table 2:** Comparison of muscle thickness between groups.

	Emmetropic Subjects		High Myopic Subjects		Test		*p*
	Mean (mm)	SD	Mean (mm)	SD			
Medial Rectus Muscle	3.50	0.50	5.09	0.57	Z	−6.07	0.00 * ES = 0.98
Lateral Rectus Muscle	3.49	0.49	4.18	0.94	Z	−2.88	0.00 * ES = 0.98
Superior Rectus Muscle	3.19	0.46	3.89	0.64	Z	−3.98	0.00 * ES = 0.67
Inferior Rectus Muscle	3.20	0.51	3.72	0.75	Z	−2.51	0.01 * ES = 0.45
Temporalis Muscle	8.99	1.77	15.20	2.78	Z	−5.66	0.00 * ES = 0.92
Masseter Muscle	9.34	1.91	16.91	3.13	Z	−5.86	0.00 * ES = 0.95

* Significant difference.

**Table 3:** Correlation results in the emmetropic subject group.

Emmetropic Subjects		Axial Length Eyeball	Medial Rectus Muscle	Lateral Rectus Muscle	Superior Rectus Muscle	Inferior Rectus Muscle	Temporalis Muscle	Masseter Muscle
Axial Length Eyeball	r	-	0.30	0.15	0.00	−0.13	−0.05	0.00
	*p*		0.12	0.45	0.98	0.51	0.80	0.99
Medial Rectus Muscle	r		-	0.68	0.22	0.27	0.14	0.46
	*p*			0.00 *	0.25	0.17	0.47	0.01 *
Lateral Rectus Muscle	r			-	0.19	0.36	0.32	0.24
	*p*				0.33	0.06	0.10	0.21
Superior Rectus Muscle	r				-	0.68	0.44	0.29
	*p*					0.00 *	0.02 *	0.14
Inferior Rectus Muscle	r					-	0.60	0.32
	*p*						0.00 *	0.10
Temporalis Muscle	r						-	0.26
	*p*							0.17
Masseter Muscle	r							-
	*p*							

* Significant difference.

**Table 4:** Correlation results in the high myopic subject group.

High Myopic Subjects		Axial Length Eyeball	Medial Rectus Muscle	Lateral Rectus Muscle	Superior Rectus Muscle	Inferior Rectus Muscle	Temporalis Muscle	Masseter Muscle	Refractive Error	Visual Acuity
Axial Length Eyeball	r	-	−0.09	−0.43	−0.25	0.14	−0.26	−0.14	−0.80	0.12
	*p*		0.68	0.04 *	0.23	0.52	0.21	0.50	0.00 *	0.67
Medial Rectus Muscle	r		-	0.58	−0.14	0.10	−0.12	−0.16	−0.16	−0.17
	*p*			0.00 *	0.53	0.65	0.59	0.47	0.57	0.57
Lateral Rectus Muscle	r			-	0.09	0.06	0.40	0.17	0.03	0.06
	*p*				0.68	0.79	0.05	0.43	0.90	0.84
Superior Rectus Muscle	r				-	−0.05	0.05	−0.20	0.28	−0.30
	*p*					0.83	0.82	0.34	0.31	0.30
Inferior Rectus Muscle	r					-	0.16	−0.04	−0.09	−0.67
	*p*						0.46	0.86	0.76	0.01 *
Temporalis Muscle	r						-	0.46	0.10	0.18
	*p*							0.02 *	0.71	0.54
Masseter Muscle	r							-	−0.26	0.49
	*p*								0.34	0.07
Refractive Error	r								-	0.26
	*p*									0.40
Visual Acuity	r									-
	*p*									

* Significant difference.

## Data Availability

Not applicable.
